# Acupuncture and physical exercise for affective symptoms and health-related quality of life in polycystic ovary syndrome: secondary analysis from a randomized controlled trial

**DOI:** 10.1186/1472-6882-13-131

**Published:** 2013-06-13

**Authors:** Elisabet Stener-Victorin, Göran Holm, Per Olof Janson, Deborah Gustafson, Margda Waern

**Affiliations:** 1 Institute of Neuroscience and Physiology, Department of Physiology, Sahlgrenska Academy, University of Gothenburg, Gothenburg, Sweden; 2 Department of Obstetrics and Gynecology, First Affiliated Hospital, Heilongjiang University of Chinese Medicine, Harbin, China; 3 Institute of Medicine, Department of Metabolism and Cardiovascular Research, Sahlgrenska Academy, University of Gothenburg, Gothenburg, Sweden; 4 Institute of Clinical Science, Department of Obstetrics and Gynaecology, Sahlgrenska Academy, University of Gothenburg, Gothenburg, Sweden; 5 Institute of Neuroscience and Physiology, Department of Psychiatry and Neurochemistry, Sahlgrenska Academy, University of Gothenburg, Gothenburg, Sweden; 6 State University of New York – Downstate Medical Center, Brooklyn, NY, USA

**Keywords:** Acupuncture, Anxiety, Depression, Exercise, Health-related quality of life, Polycystic ovary syndrome

## Abstract

**Background:**

Women with polycystic ovary syndrome (PCOS) have symptoms of depression and anxiety and impaired health related quality of life (HRQoL). Here we test the post-hoc hypothesis that acupuncture and exercise improve depression and anxiety symptoms and HRQoL in PCOS women.

**Methods:**

Seventy-two PCOS women were randomly assigned to 16 weeks of 1) acupuncture (n = 28); 2) exercise (n = 29); or 3) no intervention (control) (n = 15). Outcome measures included: change in Montgomery Åsberg Depression Rating Scale (MADRS-S), Brief Scale for Anxiety (BSA-S), Swedish Short-Form 36 (SF-36), and PCOS Questionnaire (PCOSQ) scores from baseline to after 16-week intervention, and to 16-week post-intervention follow-up.

**Results:**

A reduction in MADRS-S and BSA-S from baseline to 16-weeks post-intervention follow-up was observed for the acupuncture group. The SF-36 domains role physical, energy/vitality, general health perception and the mental component of summary scores improved in the acupuncture group after intervention and at follow-up. Within the exercise group the role physical decreased after treatment, while physical functioning and general health perception scores increased at follow-up. The emotion domain in the PCOSQ improved after 16-weeks of intervention within all three groups, and at follow-up in acupuncture and exercise groups. At follow-up, improvement in the infertility domain was observed within the exercise group.

**Conclusion:**

There was a modest improvement in depression and anxiety scores in women treated with acupuncture, and improved HRQoL scores were noted in both intervention groups. While not a primary focus of the trial, these data suggest continued investigation of mental health outcomes in women treated for PCOS.

**Trial registration number:**

ClinicalTrials.gov Identifier: NCT00484705

## Background

Polycystic ovary syndrome (PCOS) is a complex endocrine and metabolic disorder with an estimated prevalence of 8–18% depending on diagnostic criteria [1]. The characteristics of PCOS include polycystic ovaries, hyperandrogenism, irregular menstrual cycles, and metabolic abnormalities such as hyperinsulinemia and obesity [2]. PCOS adversely affects health-related quality of life (HRQoL) [3-5] and increases risk of depression and anxiety [6-13]. The recent consensus report on women’s health aspects of PCOS recommended that psychological issues be considered in all women with PCOS, and highlighted the need for development of appropriate interventions [14].

The management of PCOS is directed toward improving HRQoL by alleviating co-morbid psychiatric symptoms and preventing long-term physical and psychiatric complications [4]. First line treatment of women with PCOS is lifestyle intervention focusing on diet and exercise. In two studies, HRQoL measured by the polycystic ovary syndrome questionnaire (PCOSQ) improved across all domains after a lifestyle modification program [15,16]. In obese and overweight PCOS women, diet plus exercise and diet alone decreased depression scores and improved the PCOSQ domains of emotion, body weight, and menstrual problems [17].

Acupuncture with manual stimulation [18,19] and electrical stimulation of low-frequency, i.e. electro-acupuncture has been shown to be effective in the treatment of major depression disorder in women without PCOS [20-23], and in women with depression during pregnancy [24,25] and post partum [26]. In women with PCOS, both a standardized acupuncture protocol with manual and electrical stimulation of low-frequency and physical exercise has been demonstrated to result in more regular menses and decrease hyperandrogenemia [27]. Whether acupuncture also improves symptoms of anxiety and depression and/or HRQoL in these women has not been investigated. In women with breast cancer, 12 weeks of acupuncture improved HRQoL and sleep assessed with the Women’s Health Questionnaire [28], general well-being assessed with the Symptom Checklist and mood assessed with the Mood Scale [29]. Similarly, acupuncture improved HRQoL assessed by the short-form 36 (SF-36) in patients with chronic pain conditions, such as dysmenorrhea [30] and pain from osteoarthritis [31].

Trials of physical exercise for the treatment of depression suggest that exercise is effective for decreasing depressive symptoms, although one recent large randomized controlled trials (RCT) reported no significant effect on depressive symptoms [32,33].

Because PCOS is a chronic disease that is often accompanied by symptoms of anxiety and depression and impaired HRQoL, with health implications across the lifespan, there is a need to evaluate treatment options that have few negative side effects, such as acupuncture and physical exercise. In this study, we tested the secondary hypothesis that affective symptoms and impaired HRQoL can be improved by acupuncture and physical exercise in women with PCOS.

## Methods

### Study design

The study is a secondary analysis of a prospective randomized clinical trial (RCT) [27] in women with PCOS. All participants were Swedish women living in Gothenburg. The study was conducted at Sahlgrenska Academy at the University of Gothenburg, performed in accordance with Declaration of Helsinki, and approved by the Ethics Committee at the University of Gothenburg. The Clinical Trials Government Identifier number is NCT00484705. All participants gave written informed consent before entering the study.

### Recruitment and participants

PCOS cases were recruited between November 2005 and January 2008 by newspaper advertisements in the local community. Eligible participants had ultrasound-verified (HDI 5000, ATL, Bothell, WA) polycystic ovaries with at least 12 follicles 2–9 mm and/or an ovarian volume ≥10 ml in one or both ovaries, together with oligo/amenorrhea and/or clinical signs of hyperandrogenism. Hyperandrogenism was defined as either; hirsutism with a Ferriman Gallwey (FG) score ≥8, and/or the presence of acne as defined by responding ‘yes’ to the question, *Do you have acne?* together with circulating total testosterone ≥ 1.8 nmol/L [34]. Oligomenorrhea was defined as an intermenstrual interval >35 days and fewer than eight menstrual bleedings in the past year. Amenorrhea was defined as no menstrual bleeding in the past 90 days.

Exclusion criteria were: age ≥38 years, any pharmacological treatment for PCOS within 12 weeks of study entry, or breast feeding within 24 weeks of study entry. Other exclusion criteria were cardiovascular disease, type 2 diabetes, and endocrine or neoplastic causes of hyperandrogenemia, including androgen-secreting tumors, Cushing’s syndrome, congenital adrenal hyperplasia, and hyperprolactinaemia. In the RCT, women were randomly allocated in a 2:2:1 ratio to low-frequency EA, physical exercise, or no active intervention (control). A 2:2:1 ratio was used to facilitate recruitment. To ensure equal proportions of age and BMI in each study arm, randomization was stratified by those variables. Within each stratum, randomization was accomplished by computer using permuted blocks of 5. Based on the aforementioned inclusion and exclusion criteria, the final study population was comprised of 72 PCOS women who were assessed at baseline, followed by randomization to acupuncture (n = 28), physical exercise (n = 29), or no intervention (n = 15) for 16 weeks [27].

### Outcome measures

Questionnaires were completed at baseline, after the 16-week treatment, and 16 weeks after the last treatment. The outcome measure assessing symptoms of depression and anxiety was the CPRS-S-A [35], from which the subscales Montgomery Åsberg Depression Rating Scale (MADRS-S) [36] and the Brief Scale for Anxiety (BSA-S) [37] were extracted. MADRS-S and BSA-S each include 9 items, of which two are present in both scales. All items are rated on a 7-point Likert scale, where 0 indicates no symptoms and 6 an extremely pathological condition. Item ratings are summed, yielding a maximum value of 54 for each scale. A symptom burden of potential clinical relevance was defined as a sum total ≥11 on each scale [10].

The outcome measures to assess HRQoL were the generic SF-36 [38] and the disease-specific Polycystic Ovary Syndrome Questionnaire (PCOSQ) [39,40]. SF-36 has 36 items, of which 35 are grouped into eight domains: 1) physical function, 2) role physical, 3) role emotional, 4) social functioning, 5) mental health, 6) energy/vitality, 7) bodily pain, and 8) the experience of change in general health during the last year. The eight domains are scored from 0 (worst health) to 100 (best health). The Swedish version of the PCOSQ has been demonstrated to be valid and reliable [40]. It consists of 26 items grouped into five domains: emotions, body hair, weight concerns, infertility concerns, and menstrual irregularities. Each item is rated on a 7-point Likert scale, from 1 (poorest function) to 7 (optimal function).

### Interventions

The acupuncture treatment and physical exercise interventions have been described in detail [27]. In brief, all participants received general information about the benefits of regular exercise and were instructed to complete a physical exercise diary during weeks 1–32 of the study.

#### Acupuncture intervention

Acupuncture was given twice weekly for 2 weeks, once weekly for 6 weeks, and once every other week for 8 weeks (14 treatments over 16 weeks). Acupuncture was performed by a registered physical therapist educated in theoretical and practical acupuncture skills. Acupuncture points and electrical stimulation were the same for all women in the acupuncture group. Disposable, single-use, sterilized stainless-steel needles (Hegu Xeno, Hegu, Landsbro, Sweden; length 30/50 mm, diameter 0.32 mm) were inserted to a depth of 15–35 mm in four acupuncture points in abdominal muscles and four in the muscles below the knee, bilaterally. All points in somatic segments corresponded to the innervation of the ovaries (Th12–L2, S2–S4). Two needles were also placed in extrasegmental acupuncture points that do not innervate the ovaries (muscles in the hand and lower arm, bilaterally) to enhance central nervous system effects. All needles were stimulated manually once when inserted. During each treatment, needles in abdominal and leg muscles were stimulated electrically with low-frequency (2 Hz) (CEFAR ACUS 4, Cefar-Compex Scandinavia, Malmö, Sweden) for 30 min. The intensity was adjusted to produce local muscle contractions without pain or discomfort. Needles in the hand/lower arm were stimulated manually by rotating the needle to evoke needle sensation every 10 min.

#### Physical exercise intervention

The physical exercise program consisted of 16 weeks of regular exercise, including brisk walking, cycling, or any other aerobic exercise at a self-selected pace described as “faster than normal walking” that could be sustained for at least 30 min at least 3 days per week according to the protocol by Randeva et al. [41]. Physical exercise was self-monitored with a heart rate monitor (ECG2, Sports Instruments, US) to ensure a heart rate ≥120 beats/min. After the initial instructions of exercise, participants did not receive any direct contact with the investigators except that they were supervised through weekly telephone calls to provide guidance on how to increase physical exercise. All exercise was in addition to usual daily physical activity.

#### Controls

Like the other participants, women in the no intervention group received oral information about the benefits of regular physical exercise. All participants could call the study coordinator at any time.

### Statistical analyses

Values are presented as mean ± SD. All scores were treated as ordinal variables and assessed with nonparametric statistical tests. Sample size calculations for the RCT have been described previously [27]. Data were analyzed according to the intention to treat principle. Missing data were replaced by carrying forward the last observation to evaluate changes over baseline to after 16-week intervention, and to 16-week post-intervention follow-up i.e. 32 weeks.

Between group differences in MADRS-S, BSA-S, SF-36 and PCOSQ scores from baseline to after 16-week intervention, and to 16-week post-intervention follow-up were analyzed with the Kruskal-Wallis test followed by the Mann–Whitney *U* test. Potential within group changes (baseline versus week 16 and baseline versus week 32) were analyzed with the Wilcoxon signed-rank test*.*

IBM SPSS Statistics version 19 for Windows (SPSS, Chicago, IL) was used for statistical analyses; *P* < 0.05 (two-sided) was considered statistically significant.

## Results

Table 1 shows anthropometry and scores on PCOSQ domains for all participants. There were no differences in anthropometric measurements before and after treatment, as reported [27].

**Table 1:** Anthropometry and PCOSQ domains of women with polycystic ovary syndrome

	**PCOS (n = 72)**
Anthropometry	
Age (years)	29.9 ± 4.4
BMI (kg/m^2^)	28.1 ± 7.4
WHR	0.84 ± 0.07
PCOSQ	
Emotions	4.4 ± 1.2
Body hair	3.4 ± 2.0
Body weight	3.6 ± 2.0
Infertility	4.0 ± 1.7
Menstruation	4.0 ± 1.1

Values are mean ± SD. BMI, body mass index; WHR, waist hip ratio; PCOSQ, polycystic ovary syndrome questionnaire.

*Symptoms of depression and anxiety.* There were no baseline differences between the groups regarding anxiety and depression scores; MADRS-S and BSA-S (Table 2). Over the 16 week intervention, there was neither within group differences nor between group differences regarding change in MADRS-S or BSA-S scores (Table 3). At week 32, the MADRS-S score was significantly lower in the acupuncture group (Table 4). No change was observed between baseline and week 32 in MADRS score was observed in the exercise group at week 32. The BSA-S score was significantly lower within the acupuncture group at week 32 and differed from that in the exercise group but not the control group (Table 4). Although there was no within group change in the exercise group, the BSA-S score differed from that in the control group at week 32 (Table 4).

**Table 2:** Baseline characteristics

	**Acupuncture (n = 28)**	**Physical exercise (n = 29)**	**Control (n = 15)**	***P *Between group**
Depression & Anxiety scores				
MADRS-S sum total	10.7 ± 6.2	10.8 ± 9.0	13.8 ± 9.4	ns
BSA-S sum total	13.1 ± 6.4	11.3 ± 6.0	14.6 ± 5.7	ns
SF36 domains				
Physical functioning	88.6 ± 16.4	87.9 ± 15.0	92.3 ± 10.0	ns
Role physical	67.9 ± 33.9	86.2 ± 22.7	76.7 ± 30.6	ns
Role emotional	52.4 ± 43.9	47.1 ± 42.3	37.8 ± 41.5	ns
Social functioning	74.6 ± 21.6	67.2 ± 26.2	67.5 ± 31.3	ns
Mental health	60.3 ± 19.5	60.3 ± 19.9	56.0 ± 23.1	ns
Energy/vitality	44.3 ± 22.0	48.8 ± 23.9	46.6 ± 19.2	ns
Bodily pain	73.4 ± 19.4	71.9 ± 32.6	72.9 ± 23.5	ns
General health perception	56.6 ± 25.2	63.7 ± 18.0	64.6 ± 18.6	ns
SF36 summary scores				
Physical component summary	50.1 ± 9.8	53.1 ± 7.4	54.4 ± 6.1	ns
Mental component summary	35.7 ± 14.2	33.8 ± 12.9	30.8 ± 15.2	ns
PCOSQ				
Emotions	4.7 ± 1.2	4.2 ± 1.1	4.2 ± 1.2	ns
Body hair	4.0 ± 2.2	2.9 ± 1.9	3.3 ± 1.9	ns
Body weight	4.1 ± 2.2	3.3 ± 2.0	3.1 ± 1.7	ns
Infertility	4.2 ± 1.7	3.6 ± 1.6	4.5 ± 1.6	ns
Menstruation	4.1 ± 0.9	3.9 ± 1.2	4.0 ± 1.4	ns

Values are mean ± SD. Between-group differences at baseline were determined with the Kruskal-Wallis test.

**Table 3:** Changes in outcome measures from baseline to week 16 directly after treatment

	**Acupuncture (n = 28)**		**Physical exercise (n = 29)**		**Control (n = 15)**		P*
	**Mean ± SD**	**Δ%**	**Mean ± SD**	**Δ%**	**Mean ± SD**	**Δ%**	
Depression & Anxiety scores							
MADRS-S sum total	−0.96 ± 5.25	−9.0	0.52 ± 7.62	4.8	1.53 ± 5.33	11.1	ns
BSA-S sum total	−1.61 ± 4.72	−12.3	0.41 ± 5.62	3.6	−0.13 ± 3.96	−0.9	ns
SF-36 domains							
Physical functioning	−1.7 ± 12.9	−1.9	0.9 ± 10.2	1.0	−4.0 ± 8.1	−4.3	ns
Role physical	8.0 ± 31.2^A,a^	11.8	−16.4 ± 36.8^d^	−19.0	−6.7 ± 27.5	−8.7	**0.046**
Role emotional	6.5 ± 35.8	12.4	5.7 ± 45.5	12.1	15.6 ± 35.3	41.3	ns
Social functioning	8.0 ± 17.7 ^a^	10.7	3.0 ± 23.1	4.5	−3.3 ± 18.0	−4.9	ns
Mental health	5.7 ± 16.0	9.5	−1.1 ± 19.8	−1.8	−4.5 ± 13.7	−8.0	ns
Energy/vitality	8.4 ± 16.9 ^b^	19.0	0.7 ± 16.8	1.4	−1.2 ± 16.4	−2.6	ns
Bodily pain	2.2 ± 21.2	3.0	1.3 ± 30.2	1.8	−3.0 ± 19.7	−4.1	ns
General health perception	5.9 ± 13.6^a^	10.4	1.2 ± 11.6	1.9	−5.5 ± 16.8	−8.5	ns
SF-36 summary scores							
Physical component	0.4 ± 6.4	0.8	−1.6 ± 6.2	−3.0	−3.2 ± 4.6	−5.9	ns
Mental component	4.3 ± 9.7^b^	12.0	1.6 ± 10.5	4.7	1.5 ± 9.2	4.9	ns
PCOSQ domains							
Emotions	0.8 ± 0.8^c^	17.0	0.8 ± 1.2^e^	19.0	0.7 ± 0.7^g^	16.7	ns
Body hair	−0.1 ± 0.8	−2.5	0.1 ± 0.6	3.4	0.2 ± 1.1	6.1	ns
Body weight	0.2 ± 0.5	4.9	0.1 ± 1.1	3.0	0.6 ± 1.0 ^f^	19.4	ns
Infertility	0.3 ± 1.0	7.1	0.4 ± 1.0^d^	11.1	0.0 ± 0.7	0.0	ns
Menstruation	0.4 ± 1.0	9.8	0.4 ± 1.0	10.3	0.4 ± 0.8^f^	10.0	ns

*Intergroup differences for the changes from baseline to week 16 were determined by the Kruskal-Wallis test followed by Mann–Whitney *U*-test: ^A^*P <* 0.05 vs physical exercise. Within group changes were determined by Wilcoxon rank-sum test: ^a^*P <* 0.05 (acupuncture group); ^b^*P <* 0.01 (acupuncture group); ^c^*P <* 0.001 (acupuncture group); ^d^*P <* 0.05 (exercise group); ^e^*P <* 0.001 (exercise group); ^f^*P* < 0.05 (control group); ^g^*P* < 0.001 (control group).

**Table 4:** Changes in outcome measures from baseline to follow-up week 32

**Outcome measure**	**Acupuncture (n = 28)**		**Physical exercise (n = 29)**		**Control (n = 15)**		P*
	**Mean ± SD**	Δ**%**	**Mean ± SD**	Δ**%**	**Mean ± SD**	Δ**%**	
Depression & Anxiety scores							
MADRS-S sum total	−1.00 ± 8.07 ^a^	−9.3	0.55 ± 8.00	5.1	1.00 ± 6.94	7.2	ns
BSA-S sum total	−1.74 ± 7.88 ^A,a^	−13.3	1.41 ± 6.86 ^B^	12.5	−1.53 ± 3.78	−10.5	**0.027**
SF-36 domains							
Physical functioning	−2.5 ± 17.4	−2.8	3.8 ± 6.7 ^C,d^	4.3	−3.7 ± 7.4	−4.0	**0.008**
Role physical	12.5 ± 33.7^a^	18.4	−0.9 ± 19.5	−1.0	−6.7 ± 27.5	−8.7	ns
Role emotional	11.9 ± 40.8	22.7	17.2 ± 42.4	36.5	22.2 ±34.9 ^f^	58.7	ns
Social functioning	5.8 ± 20.5	7.8	5.2 ± 25.3	7.7	2.5 ± 21.2	3.7	ns
Mental health	6.3 ± 19.5	10.4	4.1 ± 20.0	6.8	−4.3 ± 21.9	−7.7	ns
Energy/vitality	11.3 ± 17.0^b^	25.5	5.5 ± 20.5	11.3	−4.2 ± 18.3	−9.0	ns
Bodily pain	2.8 ± 20.6	3.8	3.6 ± 30.0	5.0	0.3 ± 15.8	0.4	ns
General health perceptions	6.8 ± 16.0^a^	12.0	5.3 ± 10.0 ^c^	8.3	−4.6 ± 19.2	−7.1	ns
SF-36 summary scores							
Physical component summary	0.5 ± 7.9	1.0	0.2 ± 5.2	0.4	−1.7 ± 5.1	−3.1	ns
Mental component summary	5.1 ± 12.4^a^	14.3	4.3 ± 11.6	12.7	1.8 ± 11.6	5.8	ns
PCOSQ domains							
Emotions	0.4 ± 0.8^a^	8.5	0.6 ± 1.0 ^d^	14.3	0.1 ± 0.7	2.4	ns
Body hair	−0.1 ± 0.9	−2.5	0.1 ± 0.8	3.4	−0.2 ± 0.8	−6.1	ns
Body weight	0.2 ± 0.8	4.9	0.2 ± 1.2	6.1	0.4 ± 0.8 ^f^	12.9	ns
Infertility	0.2 ± 1.1	4.8	0.6 ± 1.2 ^B,d^	16.7	−0.1 ± 0.8	−2.2	**0.014**
Menstruation	0.3 ± 1.0	7.3	−0.1 ± 1.0	−2.6	−0.5 ± 1.0	−12.5	ns

*Intergroup differences for the changes from baseline to week 16 were determined by the Kruskal-Wallis test followed by Mann–Whitney *U*-test: ^A^*P <* 0.05 *vs* physical exercise; ^B^*P <* 0.05 *vs* control group; ^C^*P* < 0.05 vs EA and control group. Within group changes were determined by Wilcoxon rank-sum test: ^a^*P <* 0.05 (acupuncture group); ^b^*P <* 0.01 (acupuncture group); ^c^*P <* 0.05 (exercise group); ^d^*P <* 0.01 (exercise group); ^e^*P <* 0.001 (exercise group); ^f^*P* < 0.05 (control group).

*HRQoL.* There were no baseline differences between the groups regarding HRQoL; SF-36 and PCOSQ scores (Table 2). Between baseline and week 16, the SF-36 domain role physical score increased within the acupuncture group and the delta change differed from that in the exercise group which had decreased but not the control group (Table 3). The SF-36 domains social functioning, energy/vitality, general health perception and the mental component summary score increased within the acupuncture group between baseline and week 16, although there were no between group differences. Between baseline and follow up, the SF-36 domains role physical, energy/vitality, general health perception and the mental component summary score increased within the acupuncture group, with no between group differences (Table 4).

Within the physical exercise group the physical functioning score was higher at week 32 compared to baseline, and delta change differed from that in the acupuncture and control groups (Table 4). The general health perception score increased within the exercise intervention group. Within the control group, role emotional score was greater at week 32 compared to baseline.

There were no between group differences on delta change between baseline and week 16 in any of the five PCOSQ domains (Table 3). The emotions domain increased significantly within all three groups between baseline and week 16; the infertility domain increased within the exercise group, and the body weight and menstruation domains increased within the control group (Table 3).

At 32 week follow-up, improvement in the infertility domain was observed within the exercise group and delta change differed from that in the control group (Table 4). The emotion domain was higher at week 32 compared to baseline within the acupuncture and exercise groups, and the body weight domain was higher in the control group at week 32 compared to baseline.

## Discussion

This is the first study to evaluate the effect of acupuncture on affective symptoms and HRQoL in women with PCOS. We demonstrated a within group reduction in depression and anxiety scores at follow up 16 weeks after the last treatment. HRQoL was greater in the acupuncture group, as reflected by increased scores in the SF-36 domain role physical after a 16 week intervention. In addition, the SF-36 domains social functioning, energy/vitality, and general health perception and the mental component of SF-36 summary scores improved within the acupuncture group although there were no between-group differences. The effect remained at 32 weeks follow-up in the domain roles physical, energy/vitality, and general health perception and in the mental component of SF-36 summary scores. The emotion domain in the PCOSQ also improved significantly after the 16 week acupuncture intervention, which persisted at 32 weeks.

The results in the present study are in line with previous reports indicating that acupuncture can reduce symptoms of anxiety [42] and depression [22,23,25] and improve HRQoL [28,30,31] in other conditions than PCOS. The effect of acupuncture was more pronounced on anxiety symptoms and there was a clear tendency of decrease within the acupuncture group immediately after the treatment period but it did not reach statistical significance. At the follow up, the decrease was even larger and decreased symptoms of anxiety as compared to the exercise group. This may reflect that the effect of treatment last at least 4 months after the treatment period.

The acupuncture protocol used in the present study was designed to improve reproductive and endocrine function. Accordingly, needles were placed in abdominal and leg muscles with the same innervation as the ovaries. Additionally two points, one in each hand was placed. Points in arm/hand and leg/feet are considered to have a strong input to central nervous system [43]. If the primary aim of this study would have been to elucidate the efficacy of acupuncture on symptoms of anxiety and depression and HRQoL, the acupuncture protocol would have been the same but with additional points in the head, e.g. EX1 and GV20 and stimulated electrically since these points has been shown to relive symptoms of depression [22,23,25].

There was no effect on symptoms of anxiety and depression in the exercise group, while the SF-36 domain physical functioning score and the PCOSQ domain infertility improved at the 16-week follow-up compared with the control group. Directly after the treatment period, the exercise group improved in the PCOSQ domains emotion and infertility scores. The improvement in the infertility score in the exercise group may reflect an effect on menstrual function. In the primary study [27], the menstrual function was increased both after the treatment and at follow up which most likely reflect improved infertility score on PCOSQ.

A lifestyle program, including dietary intervention leading to considerable weight loss, has been shown to induce major improvement in HRQoL in patients with type 2 diabetes, and the addition of exercise was important for maintaining better HRQoL [44]. However, studies of lifestyle intervention in women with PCOS have not shown that exercise reduces symptoms of anxiety and depression and HRQoL beyond the reductions observed after dietary intervention and weight loss [15,17]. Evidently, weight loss is an important predictor of improvement in affective symptoms and HRQoL. However, the effect on HRQoL in the current study cannot be attributed to weight reduction as there were no changes in weight or BMI [27].

A recent systematic review found strong evidence that exercise reduces depressive symptoms among patients with chronic illness [45]. However, recently a large RCT including 361 adults with symptoms of depression found no effect of physical activity including three face to face sessions and 10 telephone calls over eight months [33]. The relatively weak response in the exercise group in the present study may reflect that exercise was self-directed but with telephone support. Again, these findings indicate the need for further research on the effects of exercise in women with PCOS. Structured, supervised exercise may improve affective symptoms and HRQoL and is recommended in future studies.

The PCOSQ domains, emotions, body weight, and menstruation were higher in the control group after treatment, and the body weight domain persisted higher at the week 32 follow-up. These results suggest that merely entering a study and being examined is positive. Importantly, information on the benefits of regular physical exercise and information about their condition that may have made them feel less different and/or increased their understanding of their health concerns, was provided to all women at the baseline visit.

### Methodological considerations

A strength of this study is the randomized design. However, it was also limited by the multiple comparisons and characteristics of the study design, as extensively discussed elsewhere [27,46]. Importantly, the results presented in this paper stem from a secondary analysis; the study was not designed or powered to specifically address these research questions. We suggest that future RCTs for women with PCOS include assessments of anxiety and depression symptoms and HRQoL to elucidate the impact of any intervention on these variables.

## Conclusions

In conclusion, there was a modest improvement in depression and anxiety scores in women treated with acupuncture, and improved HRQoL scores were noted in both intervention groups. Acupuncture and physical exercise are well-tolerated and safe. While not a primary focus of the clinical trial, these data suggest continued investigation of mental health outcomes in women treated for PCOS.

## Competing interests

The authors have nothing to disclose and no competing financial interest exist.

## Authors’ contributions

Conceived and designed the trial: ESV, GH, and POJ. Performed the trial: ESV, GH, and POJ. Analyzed the data: ESV, MW, and DG. Manuscript drafting and critical discussion: ESV, GH, POJ, DG, and MW**.** All authors read and approved the final manuscript.

## Pre-publication history

The pre-publication history for this paper can be accessed here:

http://www.biomedcentral.com/1472-6882/13/131/prepub
